# Rational Design of Protein Stability: Effect of (2S,4R)-4-Fluoroproline on the Stability and Folding Pathway of Ubiquitin

**DOI:** 10.1371/journal.pone.0019425

**Published:** 2011-05-16

**Authors:** Maria D. Crespo, Marina Rubini

**Affiliations:** 1 Institute of Molecular Biology and Biophysics, ETH-Hönggerberg, Zürich, Switzerland; 2 Department of Organic Chemistry, University of Konstanz, Konstanz, Germany; Berlin Institute of Technology, Germany

## Abstract

**Background:**

Many strategies have been employed to increase the conformational stability of proteins. The use of 4-substituted proline analogs capable to induce pre-organization in target proteins is an attractive tool to deliver an additional conformational stability without perturbing the overall protein structure. Both, peptides and proteins containing 4-fluorinated proline derivatives can be stabilized by forcing the pyrrolidine ring in its favored puckering conformation. The fluorinated pyrrolidine rings of proline can preferably stabilize either a C^γ^-exo or a C^γ^-endo ring pucker in dependence of proline chirality (4R/4S) in a complex protein structure. To examine whether this rational strategy can be generally used for protein stabilization, we have chosen human ubiquitin as a model protein which contains three proline residues displaying C^γ^-exo puckering.

**Methodology/Principal Findings:**

While (2S,4R)-4-fluoroproline ((4R)-FPro) containing ubiquitinin can be expressed in related auxotrophic *Escherichia coli* strain, all attempts to incorporate (2S,4S)-4-fluoroproline ((4S)-FPro) failed. Our results indicate that (4R)-FPro is favoring the C^γ^-exo conformation present in the wild type structure and stabilizes the protein structure due to a pre-organization effect. This was confirmed by thermal and guanidinium chloride-induced denaturation profile analyses, where we observed an increase in stability of −4.71 kJ·mol^−1^ in the case of (4R)-FPro containing ubiquitin ((4R)-FPro-ub) compared to wild type ubiquitin (wt-ub). Expectedly, activity assays revealed that (4R)-FPro-ub retained the full biological activity compared to wt-ub.

**Conclusions/Significance:**

The results fully confirm the general applicability of incorporating fluoroproline derivatives for improving protein stability. In general, a rational design strategy that enforces the natural occurring proline puckering conformation can be used to stabilize the desired target protein.

## Introduction

As conformationally restricted amino acid, proline adopts numerous structural and regulatory functions in protein structures. Two of its main-chain atoms are constricted within the pyrrolidine ring which possesses an exceptional conformational rigidity [1]. In protein structures, the pyrrolidine ring can adopt two different conformations: either the C^γ^ exo pucker with the C^γ^ atom pointing away from the carbonyl group or the C^γ^ endo pucker in which the C^γ^ atom is pointing towards the carbonyl group. The pucker can be influenced by the presence of an electron-withdrawing substituent on the C^γ^ atom of the pyrrolidine ring; in this way a single conformer can be biased by stereoelectronic and steric effects [2]. The proline ring pucker exerts an effect also on the main chain torsion angles; therefore the biasing of a single conformer enables to rationally manipulate the protein backbone conformation by pre-organization, with concomitant effects on protein stability. Until now, several 4-substituted proline derivatives have been used to elucidate protein structure-function relationships in the collagen triple helix [3]–[13], as well as in synthetic protein domains [14], in substrates of cyclophilin [15] and in few different proteins [16]–[21]. Recently, Raines and coworkers provided new insights on the origin of stabilization of proteins upon proline substitutions in the collagen triple helix [2]. The authors argued that the “editing” of proline residues can decisively contribute to preorganize the conformation of a polypeptide chain and to enhance protein stability by lowering the entropic costs during protein folding. The non-natural amino acid (2S,4R)-4-fluoroproline was found to stabilize the C^γ^-exo ring pucker and favors the *trans* conformation, while (2S,4S)-4-fluoroproline prefers the C^γ^-endo ring pucker and favors the *cis* conformation [16], [22], [23]. Fluorine is the most electronegative element which exerts a strong inductive effect; therefore the installing of a fluorine atom on the C^γ^ position is expected to maximize the stabilization of the proline ring pucker due to a strong gauche effect [22], [24].

Recently, Budisa and coworkers replaced the ten proline residues in EGFP with (4S)-FPro and found that the substituted protein refolded faster relative to the wild type protein [21]. Indeed, nine out of ten proline residues in wt EGFP adopt the C^γ^-endo configuration. An accurate comparison between the X-ray structures of wt EGFP and of the fluorinated EGFP variant revealed that the improved refolding properties of the latter are mostly due to the C^γ^-endo puckering stabilizing effect exerted by (4S)-FPro. On the other hand, the attempt to incorporate the (4R)-FPro isomer resulted in the expression of irreversible unfolded inclusion bodies.

**Figure 1:**
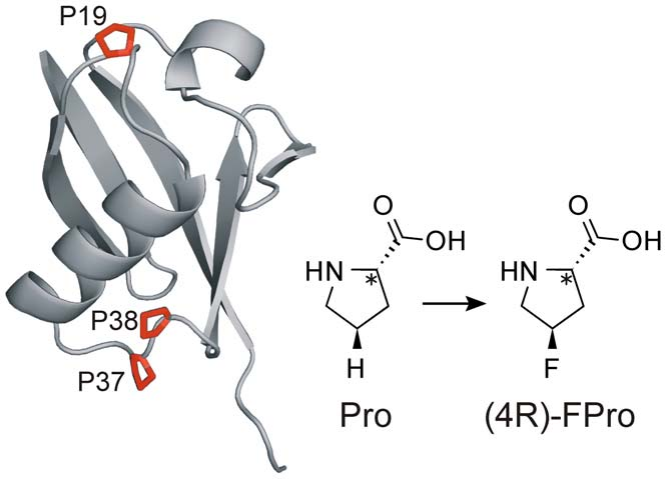
Tertiary structure of human ubiquitin according to the 1.8 Å crystal structure (1UBQ.pdb). All three proline residues (shown in red) display the C^γ^-exo conformation and were simultaneously substituted with (2S,4R)-4-fluoroproline ((4R)-FPro-ub).

Starting from this previous observation, which implies that the folding of complex proteins structures is not only determined by proline secondary structure (cis/trans isomerization) but also crucially depends on the conformation of the proline ring puckering, we decided to investigate the effect of 4-fluoroproline isomers in human ubiquitin, which contains three proline residues in its sequence. Pro 19 is located in a flexible loop connecting the N-terminal β-hairpin region to the main α-helix, while the adjacent residues Pro 37 and Pro 38 are situated in an extended loop which connects the C-terminus of the main α-helix to the β-sheets strands (Fig. 1). Ubiquitin is a highly conserved 76-residue protein of eukaryotes [25] that has been extensively characterized with respect to structure, stability, and folding dynamics [26]–[30]. Specifically, the protein shows high thermodynamic stability [31], [32] and the chemically denatured protein can be reversibly refolded *in vitro*. All three proline residues in the high-resolution X-ray structure of human ubiquitin are in the *trans* conformation and exhibit a C^γ^-exo ring pucker [33], therefore (4R)-FPro is expected to exert a beneficial effect on the stability of the protein scaffold, while (4S)-FPro should destabilize the protein. Recently, the single contributions of the proline residues in ubiquitin to its stability and folding pathway were investigated and it became evident that these residues play a significant role on protein stability, thus making it an interesting object for our studies [34].

Upon replacement of the three proline residues in human ubiquitin with 4-fluoroproline isomers we could confirm that it is possible to stabilize globular proteins by applying the pre-organization principle. Moreover, we got new insights into the influence of fluorine on the folding pathway of fluorinated proteins. The results of our study show that (4R)-FPro-ub is more stable than the wild type protein, and that the fluorinated protein folds via the same mechanism as wt-ub, while fully retaining its biological activity.

## Results

**Figure 2:**
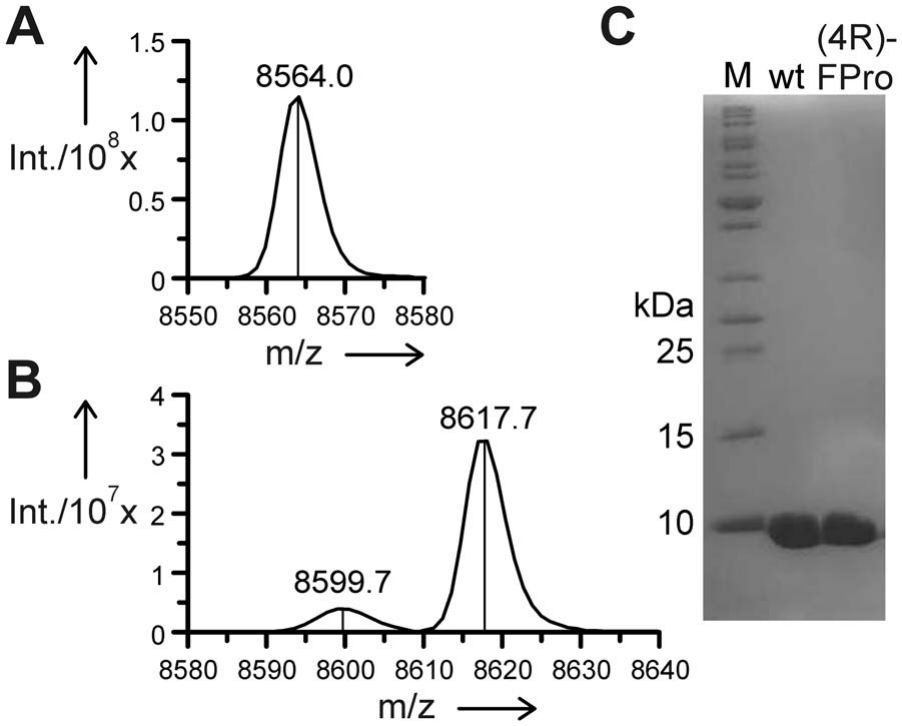
ESI-MS analyses. ESI-MS analyses of wt-ub (A), (4R)-FPro-ub (B). The minor peak corresponds to the double substituted protein. (C) wt-ub and (4R)-FPro-ub after purification on 15% SDS-PAGE.

In this study, the substitution of all three prolines with its analogs was achieved by the traditional use of the auxotrophic *E. coli* bacterial strain JM83 that is deficient in proline biosynthesis. Therefore, cells are forced to undergo selective pressure incorporation (SPI) after depletion of the natural substrate in the defined minimal medium with limiting proline concentration. ESI-MS analyses confirmed that the analog (4R)-FPro was successfully incorporated into human ubiquitin in response to the two proline codons CCG and CCT in the sequence of the ubiquitin gene. The yield of the fluorinated protein (14 mg·L^−1^) was comparable with that of wild type protein. No peak corresponding to wt-ub was detected after expression with (4R)-FPro, only a minor peak with a molecular mass of 8600 Da representing molecules with double instead of a triple (4R)-FPro incorporation was detected besides the main product peak corresponding to the all-(4R)-FPro protein (Figure 2). The attempt to incorporate (4S)-FPro failed. No discrete protein band could be detected on SDS gel after induction in the presence of (4S)-FPro, even in the sample containing the whole cell lysate, thus indicating that there is no accumulation in inclusion bodies of the substituted protein (Figure S1). The dramatic decrease of protein yield in the presence of (4S)-FPro could be explained by protein degradation due to the extreme instability of this protein variant, as confirmed by computational simulation studies.

### Structure analyses by Circular Dichroism

In order to find out if (4R)-FPro has an effect on the secondary structure of ubiquitin, far UV-CD spectrum profiles of wt-ub and (4R)-FPro-ub were recorded. The spectra of both proteins were almost identical at pH 2.0 as well as at pH 5.0 suggesting that the secondary structure of ubiquitin is not modified by incorporation of (4R)-FPro. (Figure S2)

### Thermal stability

**Figure 3:**
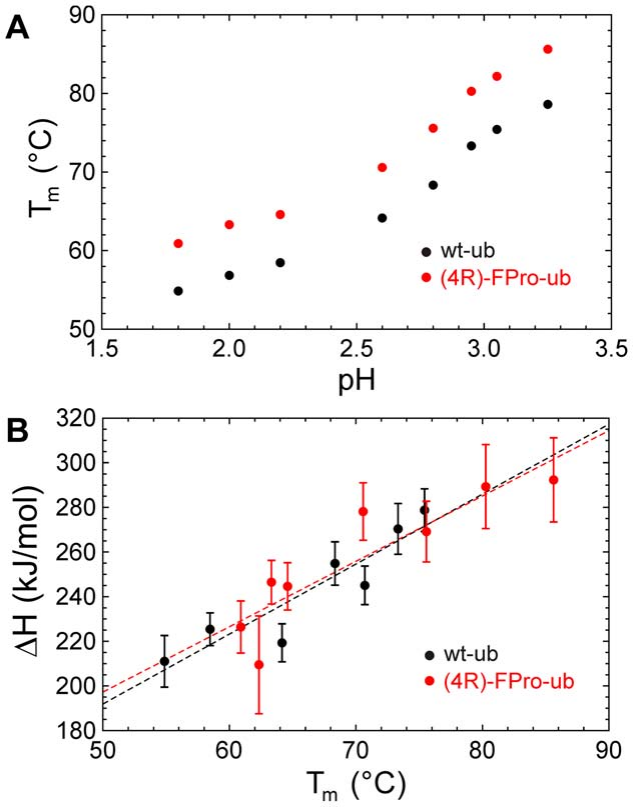
Profiles of melting temperature against pH and unfolding enthalpy against melting temperature. (A) Dependence of denaturation temperature Tm on pH for wt-ub (black circles) and (4R)-FPro-ub (red circles). (B) Unfolding enthalpy versus denaturation temperature for wt-ub (black circles) and (4R)-FPro-ub (red circles).

Ubiquitin is extremely stable at neutral pH values, exhibiting a melting point around 100°C, which complicates measurements of thermal stability at this pH. Therefore, we investigated the thermal unfolding transition of wt-ub and (4R)-FPro-ub at more acidic pH values between 1.8 and 3.25 by following the negative increase of molecular ellipticity at 200 nm. Both proteins underwent a cooperative unfolding transition. The melting points obtained for wt-ub are consistent with previous published data while the fluorinated protein displayed an increase in the melting temperature of 7°C (Figure 3a and Table 1). The plotting of ΔH_m_ values against T_m_ allows the calculation of ΔC_p_. The ΔC_p_ values obtained over the temperature ranges of 328 to 349 K for wt-ub and from 334 K to 360 K for (4R)-FPro-ub are −3.1 (±0.6) kJ·K^−1^·mol^−1^ in the case of wt-ub and −2.9 (±0.6) kJ·K^−1^·mol^−1^ for (4R)-FPro-ub (Figure 3b).

**Table 1:** Thermal unfolding parameters of wt-ub and (4R)-FPro-ub.

	wt-ub		(4R)-FPro-ub	
pH	Tm (°C)	ΔHm (kJmol^-1^)	Tm (°C)	ΔHm (kJmol^−1^)
1.8	54.9	−221	60.9	−226
2.0	56.9	−209	63.3	−246
2.2	58.5	−225	64.6	−244
2.6	64.2	−219	70.6	−278
2.8	68.3	−254	75.6	−269
2.95	73.3	−270	80.3	−289
3.05	75.4	−278	92.2	−278
3.25	78.6	−260	85.6	−292

Estimated error in T_m_ values is ±0.1°C, estimated errors in ΔHm is in the order of 5–8%.

In the literature a ΔC_p_ value close to −5 kJ·K^−1^·mol^−1^ is reported for the full U-N unfolding transition from differential scanning calorimetry analyses over a temperature range from 278 K to 398 K. The smaller temperature range in which we performed our experiments could be the reason for the deviation from the value that was previously reported.

### Effect of (2S, 4R)-4-fluoroproline on the equilibrium stability of ubiquitin

**Figure 4:**
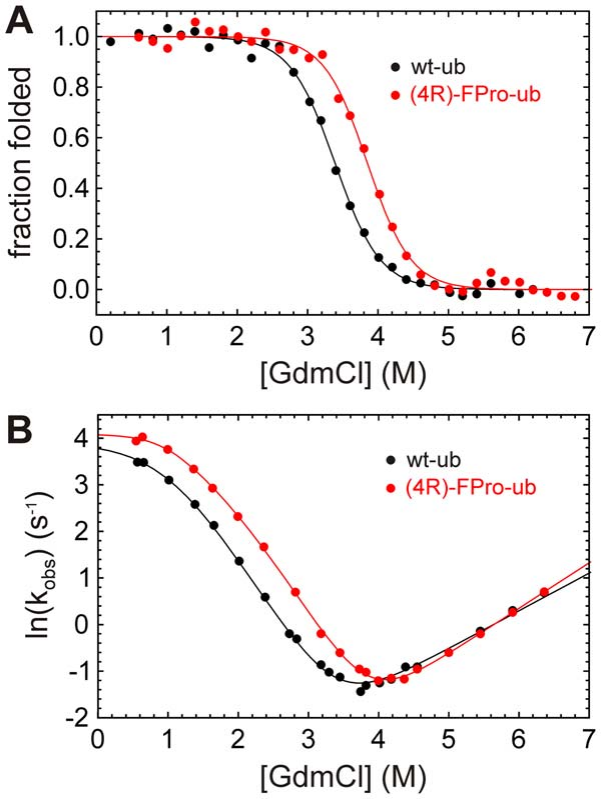
Unfolding profiles of wt-ub and (4R)-FPro-ub. (A) GdmCl-dependent equilibrium unfolding profiles of wt-ub and (4R)-FPro-ub at 25°C and pH 2.0, monitored by changes in Tyr fluorescence at 310 nm upon excitation at 278 nm. (B) Chevron plot analyses of wt-ub and (4R)-FPro-ub. Refolding and unfolding reactions were monitored by changes in the fluorescence above 300 nm of the single Tyr 59 at pH 2.0 and 25°C. Equilibrium and kinetic data were globally fitted according to a linear three-state model with a high energy intermediate. Black line (wt-ub) and red line ((4R)-FPro-ub) represent best fit to the model.

In order to test if (4R)-FPro also enhances ubiquitin stability towards chemical denaturation, we performed denaturation experiments at different concentrations of guanidinium chloride at pH 2.0 and at pH 5.0. The equilibrium stability of wt-ub and (4R)-FPro-ub was monitored by fluorescence emission at 310 nm upon excitation of tyrosine at 278 nm for measurements at pH 2.0 (Figure 4a).

The transition mid-point for wt-ub was found to be at 3.33 M GdmCl, while (4R)-FPro-ub showed a large shift in the transition mid-point to 3.78 M GdmCl. The data were globally fitted together with the unfolding and refolding kinetics data and the ΔG_un_ values were −26.16 for wt-ub and −30.87 kJ·mol^−1^ in the case of (4R)-FPro-ub. At pH 5.0 the equilibrium transitions were performed by CD measurements (Figure S3) as the fluorescence emission of tyrosine is strongly quenched by a carboxylic residue in its environment. Again, (4R)-FPro-ub appeared to be more stable in comparison to wt-ub with a transition mid-point at 4.49 M GdmCl, while the transition mid-point for the wt was found to be at 4.09 M. The ΔG_un_ values obtained from the two-state model fitting were −37.3±4.6 kJ·mol^−1^ and −33.9±3.9 kJ·mol^−1^ respectively at pH 5.0.

### Folding mechanism of (4R)-FPro-ub

To determine the influence of (4R)-FPro substitutions on the folding mechanism of ubiquitin we examined the folding/unfolding kinetics of (4R)-FPro-ub in comparison with wt-ub by stopped-flow fluorescence analysis at 25°C and pH 2.0. For both wt-ub and (4R)-FPro-ub, the refolding data were fitted according to the sum of two exponential functions (with two phases, λ_1_ and λ_2_). Specifically, only λ_1_ significantly depended on denaturant concentration. In contrast, the slower reaction λ_2_, accounting for less than 10% of the total amplitude, showed little variation upon changes in denaturant, indicative of a slow proline isomerization event, as previously reported for ubiquitin folding [27], [34]. The fact that the amplitude of λ_2_ did not change significantly in (4R)-FPro-ub indicated that the *cis*-*trans* equilibrium of Xaa-(4R)-FPro peptide bonds in the unfolded state is identical to that of Xaa-pro peptide bonds (Xaa corresponds to any amino acid). A pronounced curvature of the refolding limb in the chevron plot was observed for wt-ub and (4R)-FPro-ub at pH 2.0 at low concentrations GdmCl, indicating a deviation from two-state behavior in the folding kinetics with the formation of an early folding intermediate. Our kinetic studies show that both wt-ub and (4R)-FPro-ub fold following a sequential three-state model mechanism that involves the transient population of an obligatory on-pathway intermediate, and are fully in agreement with previously reported studies on ubiquitin folding [30], [35], [36]. The presence of intermediates in the folding mechanism of ubiquitin has been previously reported in numerous studies [27], [30], [35], [37] and in our study we observed that the intermediate for both wt-ub and (4R)-FPro-ub was populated below 1% at low denaturant concentrations. A global fit of the kinetic and equilibrium data to an on-pathway three-state model is shown in Figure 4b and data extracted from best fit is summarized in Table 2.

**Table 2:** Kinetic data for the refolding/unfolding of wt-ub and (4R)-FPro-ub monitored by changes in Tyr fluorescence using GdmCl denaturant.

	k_ui_ a	k_in/_k_iu_	k_ni_ a	m_ui_ b	m_iu_ b	m_in_ b	m_ni_ b	M_ui_ b	M_in_ b	M_un_ b	ΔG_un_ c	αTS1^d^	αTS2^e^
wt-ub	47.16	10.06	0.0103	−0.054	4.16	−1.62	2.01	4.21	3.63	7.84	−26.16	0.0129	0.447
(4R)-FPro-ub	63.64	19.76	0.0040	−0.0007	3.63	−2.10	2.41	3.63	4.51	8.14	−30.87	0.0002	0.466

Experiments were performed in 10 mM Gly/HCl buffer at pH 2.0 and at 25°C. Rate-constants and m-values listed were determined by globally fitting folding/unfolding rate constants and equilibrium fluorescence signals to a three-state on-pathway mechanism as described in material and methods.

a Rate constants have units s^−1^.

b m-values have units kJ·mol^−1^·M^−1^.

c ΔG_un_ calculated using −RT ln(K_ui_(k_in_/k_ni_)); units are kJ·mol^−1^.

d αTS1 is defined as m_ui_/(m_ui_+m_iu_).

e αTS2 is defined as m_in_/(m_in_+m_ni_).

Kinetic data obtained by stopped-flow analysis showed that the increase in protein stability at zero denaturant associated with (4R)-FPro residues are manifested by slightly faster refolding kinetics and by 2.6-fold slower unfolding kinetics (Figure 4b, Table 2). The difference in the unfolding rate values becomes evident when the unfolding arms are extended to the intersection of the *y*-axis at 0 M GdmCl concentration (Figure S4). Analysis of the *m*-values obtained from global fit and calculation of α-values, reporting the relative amount of hydrophobic surface area buried in each of the two transition states (TS1, U
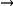
I transition, and TS2, I
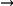
N transition, according to equation 1) revealed only small changes in the structures of TS1 and TS2 between the (4R)-FPro-ub and wt-ub (Table 2). The α-values of the first transition TS1 are close to 0 for both proteins and therefore similar to the unfolded state. The second transition in the folding mechanism (TS2) is defined by α -values of 0.44 and 0.46 for wt-ub and (4R)-FPro-ub respectively, indicating that the second transition state of wt-ub and (4R)-FPro-ub are very similar. As expected, the folding mechanism of ubiquitin was not affected by (4R)-FPro substitutions, since a sequential folding model predicts that all variants of the same protein should show a similar set of consecutive transition states [38].

### Biological activity of (4R)-FPro-ub

**Figure 5:**
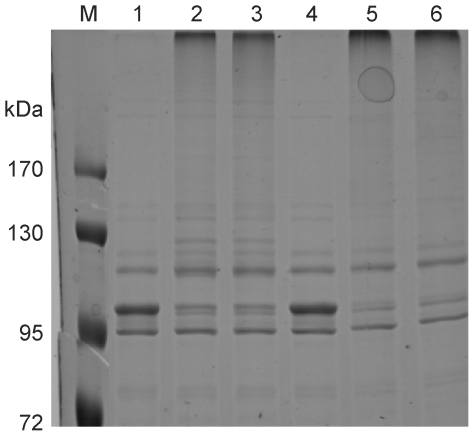
Autoubiquitination assay in the presence of E6 associating protein. After one hour of incubation, the band corresponding to the E6 associating protein (100 kDa) disappeared and was substituted by a band of higher molecular weight corresponding to the polyubiquitinated protein. M: protein marker, 1: wt-ub at t = 0 h; 2: wt-ub at t = 1 h; 3: wt-ub at t = 2 h; 4: (4R)-FPro-ub at t = 0 h; 5: (4R)-FPro-ub at t = 1 h; 6: (4R)-FPro-ub at t = 2 h.

As the incorporation of (4R)-FPro into ubiquitin may have affected the activity of the protein despite the fact that its three-dimensional structure was preserved; (4R)-FPro-ub was also subjected to a biological activity assay. Modifications of proteins by the covalent attachment of ubiquitin for proteasome-mediated degradation require the participation of several enzymes, like the ubiquitin-activating enzyme, the ubiquitin-conjugating enzyme, and the ubiquitin protein ligase. An autoubiquitination assay was performed *in vitro* in the presence of the ubiquitin-activating enzyme E1, the ubiquitin-conjugating enzyme UbcH5b, and the E6 associating protein [39], [40]. After one hour of incubation, the band corresponding to the E6 associating protein (100 kDa) disappeared and bands of higher molecular mass corresponding to polyubiquitinated E6 associating protein could be detected for both (4R)-FPro-ub and wt-ub (Figure 5). The results show that the fluorinated protein is still recognized by all these enzymes, that it can form polyubiqitin chains as wt-ub and that its biological activity is fully retained.

## Discussion

In this work we investigated the global effect of (4R)-FPro on stability and folding of human ubiquitin. As previously stated, the (4S)-FPro isomer could not be incorporated into ubiquitin, as the level of protein expression observed after induction in medium containing (4S)-FPro was extremely low (Figure S1). Since (4S)-FPro has been already successful incorporated into proteins by using auxotrophic cells, this failure cannot be attributed to the rejection of (4S)-FPro by the *E. coli* translation machineries. It seems more consistent that (4S)-FPro destabilizes the protein to a great extent thus making it subjected to protease degradation.

Our thermal denaturation studies on both wt and fluorinated protein aimed to investigate to which extent the introduction of (4R)-FPro can enhance the stability of ubiquitin. Ubiquitin is an extremely robust protein which displays a denaturation temperature even higher than 100°C at neutral pH [41], but the transition mid-point is considerably lower at acidic pH [42]. Thermal denaturation curves at all examined acidic pH values showed for (4R)-FPro-ub an increase in the melting temperature of about 7°C (Table 1), pointing towards a considerable stabilizing effect induced by fluorine. The same stabilizing effect against chemical denaturation could be observed in guanidine-induced unfolding experiments at pH 2.0 and at pH 5.0 (Table 2; Figure 4 and Figure S3). Further we performed kinetic unfolding and refolding measurements in order to examine if the presence of fluorine affects the folding mechanism of (4R)-FPro-ub. We could perform kinetics experiments only at pH 2.0, as the fluorescence emission of the single fluorescent residue in human ubiquitin, tyrosine 59, is dramatically quenched at pH 5.0. The global substitution of Pro with (4R)-FPro enhanced the stability of the protein by −4.71 kJ·mol^−1^, which is a conspicuous increase in stability for a small 76-residue protein like ubiquitin. The refolding kinetics of (4R)-FPro-ub shows a rate constant k_ui_ that is slightly faster in comparison to wt-ub, while the unfolding rate constant k_ni_ is 2.6 fold slower for the fluorinated protein (Table 2). On the protein folding pathway the formation of the intermediate seems to be favored in the case of (4R)-FPro-ub, with a faster k_ui_ and slower k_iu_ than for wt-ub. In addition, ΔG_ui_ values show that the energy barrier from U to I is lower for (4R)-FPro-ub. These results indicate that the replacement of proline with (4R)-FPro accelerates the early stages of the folding process by constraining the number of the possible conformations that can be adopted on the folding pathway. (4R)-FPro exerts a beneficial effect on the pyrrolidine ring pucker, favoring the C^γ^-exo conformation present in the wt structure, due to a stereoelectronic effect. Computer simulations were performed in order to detect potential new interactions that may arise in the protein scaffold by introduction of (4R)-FPro. The analysis of the local microenvironment of (4R)-FPro 19, (4R)-FPro 37, and (4R)-FPro 38 shows that fluorine is involved in several stabilizing interactions with amide groups of the backbone and of glutamine side chains. Besides C-F—H-N electrostatic interactions that were already identified in (4S)-FPro-EGFP, new van der Waals contacts arise in (4R)-FPro-ub in comparison to wt-ub: (4R)-FPro 19 interacts with C^ε^ of Met 1 (3.0 Å) and (4R)-FPro 38 makes a van der Waals contact with C^β^ of Lys 27 (3.2 Å) (Figure S5). All these positive interactions can be elicited only if the proline ring pucker presents the C^γ^-exo conformation. The presence of the C^γ^-endo pucker would bring the fluorine atom in a solvent exposed position with detrimental consequences for protein stability. At the same time, (4R)-FPro promotes the *trans* conformation on the peptide main chain, and it is likely that the adoption of the *trans* conformation in the course of the protein folding is energetically favored. All three proline residues in ubiquitin are located on flexible loops and are solvent exposed. Therefore, it is unlikely that the enhancement in stability derives from the self-segregating property of fluorine, as the hydrophobic core of the protein is not involved in fluorocarbon interactions. Moreover, this effect is normally elicited by incorporation of extensively fluorinated analogs of hydrophobic amino acids [43]–[48].

One of the essential roles of ubiquitin is to target proteins that have to be degraded in proteasome-mediated degradation or lysosomal degradation processes. The ubiquitin-conjugation system is highly specific and requires the intervention of several enzymes that can recognize ubiquitin only if the folding of the latter is correct. Our activity essays results indicate that monofluorination is well tolerated and does not lead to disruptions in the protein scaffold, as (4R)-FPro-ub behaves exactly as the parent protein. It has to be taken into consideration that the fluorine radius (*r*_F_ = 0.57 Å) is bigger than hydrogen (*r*_H_ = 0.31 Å) and that the C-F bond is significantly longer than the C-H bond [49]. This can result in an increase of the steric hindrance when perfluorinated amino acids are incorporated into proteins sometimes leading even to inactivation of the target protein.

In summary, our results show that the biasing of the puckering preference of proline by appropriate atomic mutations on the pyrrolidine ring can provide a new dimension in protein folding in the context of complex and rigid protein structures [50]. The spatial constriction of the substituted pyrrolidine ring favours local intramolecular interactions in the microenvironment of proline in the early stages of protein folding thus reducing the number of the possible conformations from the unfolded to the protein native state. This concept widens the role prevalently assigned to proline as secondary structure modulator (*cis/trans* isomerization) and accentuates its importance in pre-organizing the tertiary structure of globular proteins.

## Materials and Methods

### Materials

(2S,4R)-4-fluoroproline and (2S,4S)-4-fluoroproline were purchased from Bachem. All chemicals were of analytical grade and purchased from Sigma-Aldrich.

### Plasmids and strains

For incorporation experiments the proline auxotrophic *Escherichia coli* strain JM83 (F^−^ Δ(lac-proAB) w80 D(lacZ) M15 ara rpsL thi λ^−^) from ATCC (catalogue number 35607) was used. The DNA sequence corresponding to human ubiquitin was cloned from plasmid pET3a-Ub available in our laboratories into plasmid pQE-31 (Qiagen) to give the plasmid pQE-31Ub. Cells of JM83 were co-transformed with plasmid pREP4 in order to achieve tight expression regulation and plated on LB/kanamycin/carbenicillin agar plates.

### Protein expression and purification

For incorporation of proline analogs JM83/pQE-31Ub/pREP4 was grown overnight at 37°C in 20 ml of M9 minimal medium supplemented with 0.05 mM proline. The overnight culture was used to inoculate 1 L of M9 minimal medium supplemented with 0.035 mM proline, all other amino acids (50 mg/ml), thiamine (10 µg/ml), biotin (10 µg/ml) and the appropriate antibiotics (100 µg/ml carbenicillin and 35 µg/ml kanamycin). The cells were shaken until complete depletion of proline was reached. This point corresponds to a plateau in the growth curve at the optical density (OD_600_) of about 0.8. The respective proline analog was then added to a final concentration of 1 mM; cells were induced with IPTG (1 mM) and shaken overnight at 37°C. Thereafter, cells were collected by centrifugation, resuspended in 50 ml of lysis buffer (20 mM acetic acid/NaOH pH 5.0, 50 µg/ml lysozyme, 1 mM PMSF) and incubated on ice for 30 min. After sonication the lysate was put on a water bath at 75°C for 30 minutes. After centrifugation (15000 *g*, 45 min, 4°C), the supernatant was filtered and loaded onto a SP-Sepharose column, and ubiquitin was eluted with a linear NaCl gradient in 20 mM acetic acid/NaOH pH 5.0. PD 10 gel filtration columns (GE Healthcare) were used for buffer exchange for further experiments. Proteins were stored at −20°C. The purity of the proteins was analyzed by 15% SDS/PAGE and ESI-MS analysis. For wt-ub expression, bacteria were grown in rich Luria-Bertani and processed in the same manner as described. Protein concentrations were determined via protein absorbance at 280 nm, using an extinction coefficient of 0.15 mg^−1^·ml·cm^−1^. The total yield of purified protein per liter of bacterial culture was ∼30 mg for wt-ub and ∼14 mg for (4R)-FPro-ub.

### ESI-MS analyses

ESI-MS mass spectra were recorded on a Bruker Daltonics esquire 3000^+^. The protein sample was desalted to 5 mM Tris/HCl buffer pH 8.0. Samples were diluted with 2% acetic acid in water/CH_3_CN (1∶1) and were directly injected. Data was analyzed with DataAnalysis from Bruker. Calculated mass for wt ubiquitin: 8564.47 Da. Mass obtained: 8564.0 Da. Calculated mass for fluorinated ubiquitin: 8618.44 Da. Mass obtained: 8617.7 Da.

### CD Spectroscopy and fluorescence measurements

Circular dichroism spectra were recorded on a Jasco J-720 spectropolarimeter with temperature controller PFD-350S connected to the software program J-700. Denaturation curves were measured by following the molar ellipticity at 200 nm versus the temperature, with a temperature slope of 60°C/h. The wavelength at zero point of the second derivative of the melting curve was taken as melting point, T_m_. In the course of these experiments CD spectra were taken every 10 degrees between 195 and 250 nm in order to monitor the conformational changes in the secondary structure. The spectra were averaged from ten scans and the sample concentrations were 15 µM. Denaturant-induced folding transitions were followed by the decrease in ellipticity at 220 nm and by the increase/decrease in intrinsic tyrosine fluorescence. For equilibrium experiments, samples were incubated at different GdmCl concentrations for 2 h at 25°C before spectroscopic measurements. Fluorescence measurements were performed on a Hitachi F4500 fluorescence spectrometer at 25°C using an excitation slit of 2.5 nm and an emission slit of 5 nm. All samples were excited at λ = 278 nm. Fluorescence spectra were recorded at a scan speed of 60 nm/min, and fluorescence emission for equilibrium unfolding experiments was followed at λ = 310 nm. The sample concentration was kept at 5 µM.

### Stopped-flow kinetics

Stopped-flow experiments were performed by diluting unfolded protein (in 6.4 M GdmCl, 10 mM glycine/HCl, pH 2.0) or native protein (in 10 mM glycine/HCl, pH 2.0) into different concentrations of GdmCl in 10 mM glycine/HCl buffer, pH 2.0. The final protein concentration was 10 µM. The reactions were monitored by the change in fluorescence above 300 nm upon excitation at 278 nm using stopped-flow mixing in an Applied Photophysics SM-17MV instrument with a fixed mixing ratio of 1∶10. An average of at least five shots was collected for each reaction condition. Unfolding kinetics were fitted to a single-exponential function. Refolding kinetics were fitted to double-exponential function, where about 10% of the amplitude accounted for a slow refolding phase, presumably caused by *cis*-to-*trans* proline isomerisation of molecules with *cis*-prolines that accumulated during long-term incubation in concentrated GdmCl solution. Due to its small amplitude and independence to variation of GdmCl concentration, the contribution of this slow refolding phase was not included in the fitting procedure. The data from equilibrium fluorescence and stopped-flow measurements were fitted globally to a sequential mechanism with an on-pathway intermediate according to equation 1 using pro Fit 6.1.11.
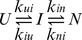
(1)


The k_iu_ and m_iu_ values were initially set to 10^4^ s^−1^ and 0 kJ·mol^−1^·M^−1^ as starting parameters, so that this step is not rate-limiting. Data analysis was performed by a weighted non-linear least-squares function using the Levenberg-Marquardt algorithm and a Monte Carlo method to fit all kinetic parameters [38], [51]. After several fittings the same ratio for k_in_/k_iu_ was obtained whereas the rest of variables reached a fixed value.

### Ubiquitin Activity Assay

The ubiquitin-activating enzyme E1, the ubiquitin-conjugating enzyme UbcH5b, and the ubiquitin-protein ligase E6-associated protein (E6-AP) were expressed in the baculovirus system as described [39], [40]. For *in vitro* ubiquitination, ubiquitin (5 µg) was incubated with 50 ng of E1, 50 ng UbcH5b and 50 ng E3 ligase in 25 mM Tris/HCl pH 7.5, 50 mM NaCl, 2 mM DTT, 4 mM ATP, 4 mM MgCl_2_ (total sample colume: 50 µl). After incubation at 30°C for 2 h, the reaction mixtures were separated *via* 15% SDS-polyacrylamide gels. The gels were stained with Roti®-Blue Colloidal Coomassie and analyzed for the disappearance of the band corresponding to E6AP and the appearance of high-molecular weight bands corresponding to polyubiquitinylated E6-AP.

## Supporting Information

Figure S1 **Expression profile of (4R)-FPro-ub and (4S)-FPro-ub.** Expression profile of (1) (4R)-FPro-ub and (2) (4S)-FPro-ub in whole cell lysate. (4R)-FPro-ub (3) and (4S)-FPro-ub (4) after sonication of the lysate and heating in water bath at 75°C for 30 minutes. M: protein marker.
(TIF)

Figure S2 **Circular dichroism spectra.** Circular dichroic spectra of wt-ub (black line) and (4R)-FPro-ub (red line) in 10 mM Glycine/HCl buffer at pH 2.0 (continuous line) and in 25 mM acetate buffer at pH 5.0 (dotted line).
(TIF)

Figure S3 **Unfolding profiles of wt-ub and (4R)-FPro-ub.** Unfolding profiles of wt-ub (black open diamond) and (4R)-FPro-ub (red open circles) by GdmCl monitored by changes in ellipticity at 220 nm. Experiments were performed at 25°C at pH 5.0 in 25 mM sodium acetate buffer.
(TIF)

Figure S4 **Chevron plot of wt-ub and (4R)-FPro-ub.** Dashed lines show the intersection of the unfolding arms with the *y*-axis at [GdmCl] = 0. The slopes are defined by y = ln k_ni_+m_ni._
(TIF)

Figure S5 **Analysis of the local microenvironment of (4R)-FPro by computational studies.** Replacement of Pro with (4R)-FPro has been simulated using the crystal structure coordinates of wt ubiquitin (PDB: 1UBQ). A run of energy minimization was performed using the SYBYL package (Tripos, Inc., St. Louis, Missouri), in order to check the potential interactions of the fluorine atom with neighbouring residues. (A) van der Waals interaction between (4R)-FPro 19 and C^ε^ of Met 1. (B) (4R)-FPro 37 interacts with the amide group in the side chain of Gln 31. (C) (4R)-FPro 38 interacts with the backbone amide group of Ala 28, with the side chain amide group of Gln 41, and it makes also a van der Waals contact with C^β^ of Lys 27.
(TIF)
